# Readmission after enhanced recovery video-assisted thoracoscopic surgery wedge resection

**DOI:** 10.1007/s00464-024-10700-6

**Published:** 2024-02-20

**Authors:** Lin Huang, Henrik Kehlet, René Horsleben Petersen

**Affiliations:** 1 Department of Cardiothoracic Surgery, Copenhagen University Hospital, Rigshospitalet, Copenhagen, Denmark; 2 Section for Surgical Pathophysiology, Copenhagen University Hospital, Rigshospitalet, Copenhagen, Denmark

**Keywords:** Enhanced recovery after surgery, Video-assisted thoracoscopic surgery, Readmission, Pulmonary wedge resection, Postoperative adverse events

## Abstract

**Background:**

Despite the implementation of Enhanced Recovery After Surgery (ERAS) programs, surgical stress continues to influence postoperative rehabilitation, including the period after discharge. However, there is a lack of data available beyond the point of discharge following video-assisted thoracoscopic surgery (VATS) wedge resection. Therefore, the objective of this study is to investigate incidence and risk factors for readmissions after ERAS VATS wedge resection.

**Methods:**

A retrospective analysis was performed on data from prospectively collected consecutive VATS wedge resections from June 2019 to June 2022. We evaluated main reasons related to wedge resection leading to 90-day readmission, early (occurring within 0–30 days postoperatively) and late readmission (occurring within 31–90 days postoperatively). To identify predictors for these readmissions, we utilized a logistic regression model for both univariable and multivariable analyses.

**Results:**

A total of 850 patients (non-small cell lung cancer 21.5%, metastasis 44.7%, benign 31.9%, and other lung cancers 1.9%) were included for the final analysis. Median length of stay was 1 day (IQR 1–2). During the postoperative 90 days, 86 patients (10.1%) were readmitted mostly due to pneumonia and pneumothorax. Among the cohort, 66 patients (7.8%) had early readmissions primarily due to pneumothorax and pneumonia, while 27 patients (3.2%) experienced late readmissions mainly due to pneumonia, with 7 (0.8%) patients experiencing both early and late readmissions. Multivariable analysis demonstrated that male gender, pulmonary complications, and neurological complications were associated with readmission.

**Conclusions:**

Readmission after VATS wedge resection remains significant despite an optimal ERAS program, with pneumonia and pneumothorax as the dominant reasons. Early readmission was primarily associated with pneumothorax and pneumonia, while late readmission correlated mainly with pneumonia.

More than two decades ago, the concept of fast-track surgery or enhanced recovery after surgery (ERAS) was introduced for reducing surgical stresses [1]. Subsequently, with implementation of ERAS programs, procedure-specific guidelines were published in different surgical specialities including lung surgery [2, 3]. However, challenges remain to achieve further accelerated rehabilitation after surgery, especially after discharge [4]. Readmission serves as a valuable metric for post-discharge evaluation in surgery.

Compared to pulmonary lobectomy, wedge resection is applied more frequently to remove or diagnose small pulmonary nodules with removal of less lung tissue [5], and the majority of patients with video-assisted thoracoscopic surgery (VATS) wedge resection following an ERAS protocol has short length of stay (LOS) [6]. While readmission rates did not significantly increase following early discharge after pulmonary lobectomy [7, 8], it is worth noting that they remained relatively high, even within the context of an ERAS VATS setting [9]. However, no procedure-specific data exist on readmissions for patients after VATS wedge resection following ERAS programs.

Therefore, this study aimed to assess the incidence and risks of readmissions after ERAS VATS wedge resection.

## Materials and methods

### Study design, setting, and data sources

This study employed a retrospective observational design, utilizing data derived from prospectively collected consecutive VATS wedge resections conducted between June 2019 and June 2022 at a high-volume university hospital.

**Fig. 1:**
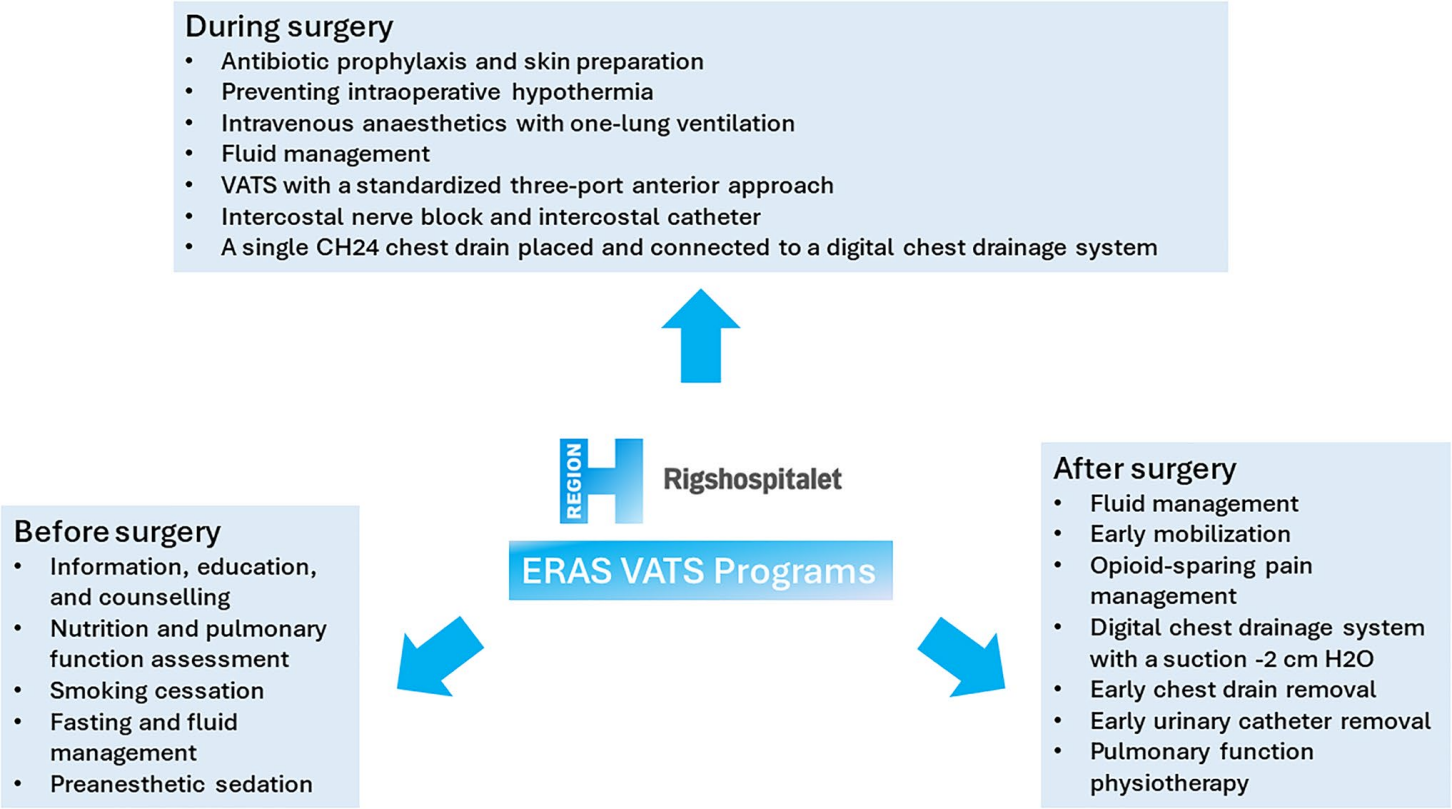
The protocol of enhanced recovery after thoracoscopic wedge resection in Rigshospitalet. *ERAS* enhanced recovery after surgery; *VATS* video-assisted thoracoscopic surgery

The Department of Cardiothoracic Surgery at Rigshospitalet, Copenhagen is the only centre in eastern Denmark and completes lung surgery mainly by a VATS procedure (> 90%, https://www.lungecancer.dk/dlcg/) with an ERAS setting. The ERAS VATS wedge resection program is similar to that of VATS lobectomy [9, 10]. All procedures were performed as a standard three-port anterior approach [11]. Intraoperatively, we used the same stapler (Medtronic, Minnesota, U.S) for all cases. All junior surgeons were supervised by a senior surgeon. The ERAS practice predominantly adheres to the current ERAS guidelines [3]. The primary elements of our ERAS VATS protocol for wedge resection are shown in Fig. 1. The early mobilization management involved encouraging patients to stand up beside the bed after 3 h postoperatively, followed by walking around the bed and to the toilet within 3–6 h postoperatively. Nurses guide all patients in respiratory physiotherapy. The single chest drain is removed when the air leak < 20 ml/h for 12 h without chyle or blood. No fixed upper limit is set for serous output. The urinary catheter is removed in the morning of postoperative day 1. The discharge criteria include self-mobilization ability, removal of all lines and the chest drain, and not requiring inpatient care.

All data were extracted from the national medical registration system (E-journal), which includes comprehensive enrolment and follow-up information for economic reimbursement purposes. This extraction process was facilitated using an electronic healthcare software program (Epic, Madison, WI, USA). Subsequently, data were stored in the Research Electronic Data Capture tool (REDCap, https://www.project-redcap.org/). Results of data analyses were reported in line with the Strengthening the Report of Observational Studies in Epidemiology (STROBE) guidelines [12].

We obtained approvals from the Danish Patient Safety Authority/the Institutional Review Board (R-22068332) and the Danish Data Protection Agency (P-2022-465) prior to start of study. The Danish health research laws and regulations waived the need for informed consent from patients due to the retrospective study design.

### Patients

Adult patients (≥ 18 years of age) who reside in eastern Denmark and underwent a VATS wedge resection were included. All participants should complete 90-day follow-up after surgery.

Patients who received pulmonary surgery within 90 days before the wedge resection, immediate anatomic pulmonary resection after frozen section pathology of wedge resection, only pleural or other biopsies replacing wedge resection, completion lobectomy after wedge resection, or death in hospital were excluded.

### Variables

Demographics included age, gender, body mass index, smoking status, alcohol status, prior lung surgery > 90 days preoperatively, and prior oncological therapy.

Clinicopathological characteristics included lung function, American Society of Anaesthesiologists classification (ASA), age adjusted Charlson Comorbidity index (CCI), surgical duration, number of wedges resected, distribution of resection, located lobe of lesion, surgeon experience, maximum dimension of wedge-resected edge, pathological diagnosis, LOS, readmissions, and morbidity and mortality.

Prior oncological therapy was defined as history of non-surgical treatment to cancer. Comorbidity and morbidity were diagnosed following the International Classification of Diseases, 10th revision (ICD-10). LOS was calculated given number of nights in hospital. As wedge resection can be employed for both diagnostic and therapeutic purposes in cases of malignant and benign conditions, subsequent programs potentially become more intricate. Thus, in this study, readmission was defined as surgical-related overnight admission to hospital.

### Outcomes

Primary outcomes were rates and reasons for 90-day readmission, early readmission (postoperative 0–30 days [POD 0–30]) and late readmission (POD 31–90). Main reasons for readmissions were evaluated. Secondary outcomes were predictors for readmissions.

### Statistical methods

The Kolmogorov–Smirnov and the Shapiro–Wilk tests showed all continuous data with non-normal distribution. Continuous data were presented as median (interquartile range [IQR]) and categorical variables as numbers (proportion). There were 2% missing data for lung function. We imputed them using the median values. Logistic regression model determined predictors for readmissions from all demographics and clinicopathological characteristics. Characteristics with *P* < 0.2 in univariable analysis were entered into multivariable analysis for identifying final predictors. Significant difference was considered as two-side *P* < 0.05. R Software (version 4.3.2, R Foundation for Statistical Computing, Vienna, Austria) was used to complete all analyses.

## Results

**Fig. 2:**
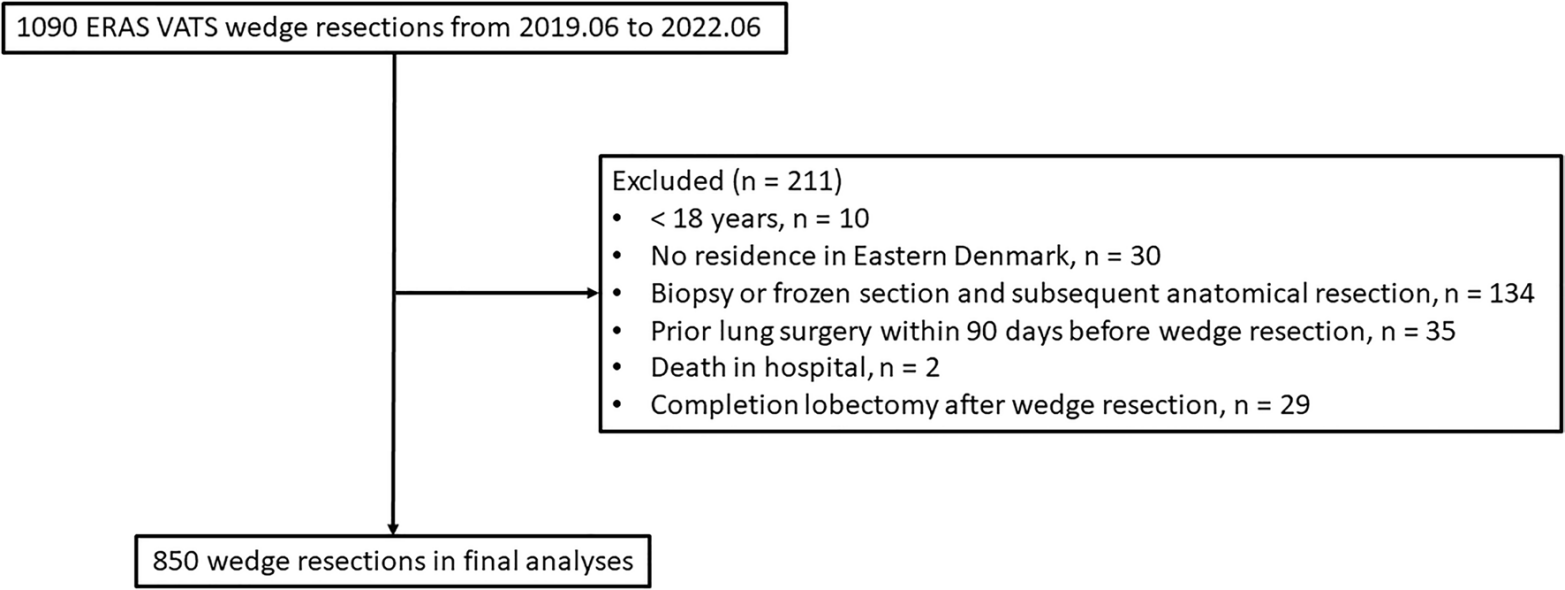
Patient enrolment for this study. *ERAS* enhanced recovery after surgery; *VATS* video-assisted thoracoscopic surgery

Of 1090 patients with ERAS VATS wedge resection, 850 were included for final analyses (Fig. 2). There were 21.5% patients (*n* = 183) with non-small cell lung cancer (NSCLC), 44.7% (*n* = 380) pulmonary metastasis, 31.9% (*n* = 271) with benign disease and 1.9% (*n* = 16) other lung cancers. Most patients (82.0%, 697/850) underwent single pulmonary wedge resection, with a median surgical duration of 38 min (IQR 28–53). There were 49.5% (*n* = 421) wedge resections performed by junior surgeons (*n* = 30) while there were 50.5% ones (*n* = 429) performed by senior surgeons (*n* = 8). Median LOS was 1 day (IQR 1–2). (Table 1 and 2).

**Table 1:** Baseline characteristics (*n* = 850)

Variables	
Age, year, median (IQR)	67 (58–75)
Gender, n (%)	
Male	439 (51.6%)
Female	411 (48.4%)
BMI, kg/m^2^, median (IQR)	25.6 (22.5–29.2)
Smoking status, n (%)	
None	266 (31.3%)
Former smoker	398 (46.8%)
Current smoker	186 (21.9%)
Alcohol abuse (> 10 items/week), n (%)	196 (23.1%)
Prior lung surgery > 90 days preoperatively, n (%)	151 (17.8%)
Prior oncological therapy, n (%)	292 (34.4%)
Lung function, median (IQR)	
FEV_1_%_pre_	93 (76–108)
FEV_1_/FVC, %	74 (66–80)
DLCO%_pre_	72 (58–85)
ASA score, n (%)	
I	2 (0.2%)
II	158 (18.6%)
III	654 (76.9%)
IV	36 (4.2%)
CCI score, median (IQR)	6 (4–7)
Surgical duration, min, median (IQR)	38 (28–53)
Number of resected pulmonary wedges, n (%)	
1	697 (82.0%)
2	124 (14.6%)
3	22 (2.6%)
4	4 (0.5%)
5	3 (0.4%)
Distribution of resection, n (%)	
Left	383 (45.1%)
Right	467 (54.9%)
Located lobe of lesion, n (%)	
Upper	367 (43.2%)
Middle	67 (7.9%)
Lower	326 (38.4%)
Upper + Middle	10 (1.2%)
Middle + Lower	21 (2.5%)
Upper + Lower	55 (6.5%)
Upper + Middle + Lower	4 (0.5%)
Surgeon experience	
Junior	421 (49.5%)
Senior	429 (50.5%)

*ASA* American Society of Anaesthesiologists classification; *BMI* body mass index; *CCI* Charlson Comorbidity index; *DLCO%*_*pre*_ percentage of predicted diffusion capacity for carbon monoxide; *FEV*_*1*_ forced expiratory volume in 1s; *FEV*_*1*_*%*_*pre*_ percentage of predicted FEV_1_; *FVC* forced vital capacity; *IQR* interquartile range

**Table 2:** Postoperative outcomes and pathological characteristics (*n* = 850)

Variables	
Pathological diagnosis, n (%)	
Benign	271 (31.9%)
NSCLC	183 (21.5%)
Metastasis	380 (44.7%)
Other lung cancers^#^	16 (1.9%)
Postoperative complications during index hospitalization, n (%)	
Pulmonary complications	60 (7.1%)
Pneumothorax	17 (2.0%)
Pneumonia	25 (2.9%)
Pleural effusion	5 (0.6%)
Oxygen dependency	36 (4.2%)
Cardiac complications	11 (1.3%)
Atrial fibrillation	11 (1.3%)
Gastrointestinal complications	36 (4.2%)
Constipation/Diarrhoea	15 (1.8%)
PONV	21 (2.5%)
Cecum volvulus	1 (0.1%)
Urinary complications	25 (2.9%)
Urinary tract infection	7 (0.8%)
Urinary retention	17 (2.0%)
Acute kidney failure	2 (0.2%)
Neurological complications	15 (1.8%)
Cognitive confusion/Delirium	13 (1.5%)
Stroke	2 (0.2%)
Postoperative bleeding	24 (2.8%)
Treated by tranexamic acid	20 (2.3%)
Treated by operation	4 (0.5%)
Wound complications	8 (0.9%)
Pain treated by opioid on POD 1	128 (15.1%)
LOS, day, median (IQR)	1 (1–2)
Duration of chest drainage, day, median (IQR)	1 (1–1)
90-day readmission, n (%)	86 (10.1%)
0–30-day readmission	66 (7.8%)
31–90-day readmission	27 (3.2%)
Time to first 90-day readmission, day, median (IQR)	13 (5–29)
Time to first 0–30-day readmission	8 (4–16)
Time to first 31–90-day readmission	40 (28–67)
LOS of 90-day readmission, day, median (IQR)	4 (2–6)
0–30-day readmission	3 (2–7)
31–90-day readmission	4 (2–6)
Mortality up to POD 90, n (%)	7 (0.8%)

#Other lung cancers included eight non-small cell lung cancer with uncertain original, two small cell lung cancer, one sarcoma, three mixed of primary non-small cell lung cancer and metastasis, one mixed of amyloid tumour and lymphoma, and one B-cell lymphoma

*IQR* interquartile range; *LOS* length of stay; *NSCLC* non-small cell lung cancer; *POD* postoperative day; *PONV* postoperative nausea and vomiting

During the index hospitalization period, the following complications were observed in patients who underwent wedge resection: pulmonary complications in 7.1% of patients (*n* = 60), cardiac complications in 1.3% of patients (*n* = 11), gastrointestinal complications in 4.2% of patients (*n* = 36), urinary complications in 2.9% of patients (*n* = 25), neurological complications in 1.8% of patients (*n* = 15), postoperative bleeding in 2.8% of patients (*n* = 24), wound complications in 0.9% of patients (*n* = 8), and postoperative pain in 15.1% of patients (*n* = 128). Seven patients (0.8%) died within 90 days postoperatively. None were related to the wedge resection however attributed to severe complications following the original cancer resection (*n* = 3), new metastasis or recurrence (*n* = 3), and suicide (*n* = 1). (Table 2).

In this cohort, 10.1% (86/850) of patients experienced readmissions within 90 days. Specifically, 7.8% (66/850) of patients experienced early readmission, while 3.2% (27/850) experienced late readmission, with 0.8% (7/850) patients experiencing both early and late readmissions. (Table 2) Furthermore, multiple readmissions were observed in seven (0.8%) patients during the early period (all experienced second early readmission) and in four (0.5%) patients during the late period (four patients experienced second late readmission and two patients experienced third late readmission).

Median time to first readmission within postoperative 90 days was 13 (5–29) days with a median length of readmission stay of 4 days (IQR 2–6). To be more specific, median time to first early readmission and first late readmission was 8 days (IQR 4–16) and 40 days (IQR 28–67), respectively. Median length of readmission stay was 3 days (IQR 2–7) from POD 0–30 and 4 days (IQR 2–6) from POD 31–90. (Table 2).

**Fig. 3:**
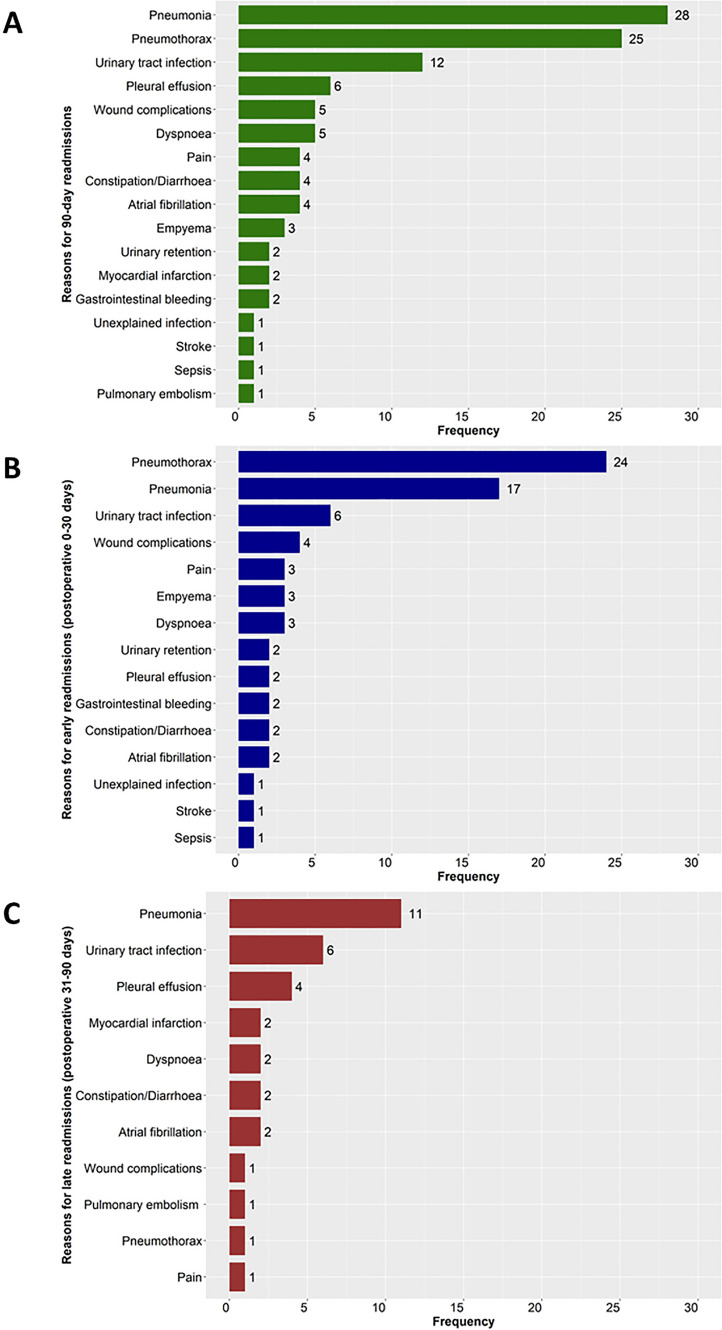
Specific reasons for readmissions after enhanced recovery thoracoscopic wedge resection. **A** reasons for readmissions during the first 90 days after surgery (*n* = 106); **B** reasons for readmissions during early period (postoperative 0–30 days) (*n* = 73); **C** reasons for readmissions during late period (postoperative 31–90 days) (*n* = 33)

The specific reasons for readmissions within the first 90 days are shown in Fig. 3A. The dominant reasons were pneumonia (28/850, 2.9%) and pneumothorax (25/850, 3.3%). Figures 3B and C display the distinct reasons behind both early and late readmissions, respectively. Pneumothorax (24/850, 2.8%) and pneumonia (17/850, 2.0%) were mainly attributed to early readmissions, while pneumonia (11/850, 1.3%) was the most important reason for late readmissions. Moreover, urinary tract infection emerged as another significant reason for readmissions, regardless of whether they occurred within the 90-day period, between 0 and 30 days, or between 31 and 90 days postoperatively.

In the multivariable analysis, male (odds ratio [OR] 1.71, 95% confidence interval [95% CI] 1.02 to 2.85, *P* = 0.036), pulmonary complications (OR 4.51, 95% CI 1.24 to 16.40, *P* = 0.022) and neurological complications (OR 3.06, 95% CI 1.30 to 7.23, *P* = 0.011) were identified as risk factors. Additionally, several parameters that did not exhibit statistical significance in the adjusted predictive model showed statistical significance in the univariable analysis. These included an increase of 10 years in age (OR 1.26, 95% CI 1.02 to 1.56, *P* = 0.030), current smoking status (OR 1.94, 95% CI 1.02 to 3.72, *P* = 0.044), alcohol abuse (OR 1.82, 95% CI 1.13 to 2.94, *P* = 0.014), a 10% increase in percentage of predicted forced expiratory volume in 1 (OR 0.85, 95% CI 0.77 to 0.94, *P* < 0.001) and percentage of predicted diffusion capacity for carbon monoxide (OR 0.85, 95% CI 0.76 to 0.95, *P* = 0.005), ASA IV classification (OR 4.11, 95% CI 1.58 to 10.70, *P* = 0.004), a 1-point increase in CCI score (OR 1.13, 95% CI 1.04 to 1.24, *P* = 0.004), a 5-min increase in surgical duration (OR 1.01, 95% CI 1.00 to 1.02, *P* = 0.009), postoperative bleeding (OR 3.11, 95% CI 1.20 to 8.06, *P* = 0.020), and a 1-day increase in LOS (OR 1.04, 95% CI 1.00 to 1.09, *P* = 0.039). (Table 3).

**Table 3:** Logistic regression analyses for patients with 90-days readmissions (*n* = 86) in the cohort (*n* = 850)

Variables	Univariable analysis		Multivariable analysis	
	OR (95% CI)	*P* value	OR (95% CI)	*P* value
Age, per 10 years increased	1.26 (1.02 to 1.56)	**0.030**	1.02 (0.79 to 1.33)	0.863
Male (Reference: Female)	1.97 (1.23 to 3.14)	**0.005**	1.71 (1.02 to 2.85)	**0.036**
BMI, per 5 kg/m^2^ increased	0.85 (0.68 to 1.05)	0.130	0.91 (0.71 to 1.16)	0.443
Smoking status (Reference: Never)				
Former smoker	1.76 (0.99 to 3.11)	0.053	1.20 (0.63 to 2.29)	0.576
Current smoking	1.94 (1.02 to 3.72)	**0.044**	1.36 (0.66 to 2.83)	0.404
Alcohol abuse (Reference: No)	1.82 (1.13 to 2.94)	**0.014**	1.41 (0.83 to 2.42)	0.208
Prior lung surgery > 90 days preoperatively (Reference: No)	1.07 (0.60 to 1.89)	0.830		
Prior oncological therapy (Reference: No)	0.86 (0.53 to 1.39)	0.543		
FEV_1_%_pre_, per 10% increased	0.85 (0.77 to 0.94)	** < 0.001**	0.91 (0.81 to 1.03)	0.140
FEV_1_/FVC, per 10% increased	0.92 (0.76 to 1.11)	0.388		
DLCO%_pre_, per 10% increased	0.85 (0.76 to 0.95)	**0.005**	0.93 (0.81 to 1.08)	0.341
ASA score (Reference: ASA I-II)				
ASA III	1.36 (0.72 to 2.59)	0.346	0.81 (0.40 to 1.64)	0.550
ASA IV	4.11 (1.58 to 10.70)	**0.004**	2.30 (0.78 to 6.77)	0.131
Charlson Comorbidity Index, per 1 increased	1.13 (1.04 to 1.24)	**0.004**	1.10 (1.00 to 1.22)	0.060
Surgical duration, per 5 min increased	1.01 (1.00 to 1.02)	**0.009**	1.01 (1.00 to 1.02)	0.127
Wedge resected > 1 (Reference: wedge resected = 1)	1.33 (0.78 to 2.29)	0.299		
Lesion on the right hemithorax (Reference: on the left hemithorax)	0.76 (0.49 to 1.19)	0.231		
Located lobe of lesion (Reference: Upper)				
Middle	1.57 (0.71 to 3.45)	0.262		
Lower	1.26 (0.76 to 2.07)	0.369		
Multiple	0.99 (0.44 to 2.22)	0.976		
Junior surgeons (Reference: senior surgeons)	0.96 (0.62 to 1.48)	0.837		
Pathological diagnosis (Reference: Benign)				
NSCLC	1.41 (0.78 to 2.58)	0.258		
Metastasis	1.00 (0.58 to 1.71)	0.995		
Other lung cancers#	2.27 (0.61 to 8.51)	0.224		
Pulmonary complications (Reference: No)	3.38 (1.79 to 6.36)	** < 0.001**	3.06 (1.30 to 7.23)	**0.011**
Cardiac complications (Reference: No)	0.89 (0.11 to 7.01)	0.910		
Urinary complications (Reference: No)	2.30 (0.84 to 6.28)	0.105	0.87 (0.24 to 3.20)	0.834
Gastrointestinal complications (Reference: No)	1.46 (0.55 to 3.86)	0.446		
Neurological complications (Reference: No)	4.65 (1.55 to 13.95)	**0.006**	4.51 (1.24 to 16.40)	**0.022**
Pain (Reference: No)	1.22 (0.67 to 2.20)	0.515		
Postoperative bleeding (Reference: No)	3.11 (1.20 to 8.06)	**0.020**	2.29 (0.74 to 7.11)	0.151
Wound complications (Reference: No)	1.27 (0.16 to 10.47)	0.823		
LOS, per 1 day increased	1.04 (1.00 to 1.09)	**0.039**	0.95 (0.88 to 1.02)	0.134
Duration of chest drainage, per 1 day increased	1.03 (0.98 to 1.09)	0.247		

*P* < 0.05 in the logistic regression analysis was shown in bold

#Other lung cancers included eight non-small cell lung cancer with uncertain original, two small cell lung cancer, one sarcoma, three mixed of primary non-small cell lung cancer and metastasis, one mixed of amyloid tumour and lymphoma, and one *B*-cell lymphoma

*ASA* American Society of Anaesthesiologists classification; *BMI* body mass index; *DLCO%*_*pre*_ percentage of predicted diffusion capacity for carbon monoxide; *FEV*_*1*_ forced expiratory volume in 1s; *FEV*_*1*_*%*_*pre*_ percentage of predicted FEV_1_; *FVC* forced vital capacity; *LOS* length of stay; *NSCLC* non-small cell lung cancer; *OR* odds ratio; *95% CI* 95% confidence interval

## Discussion

The surgical-related readmission rate serves as a crucial metric for assessing treatment quality, which provides essential evidence for policymakers, clinical leaders, and patients to make informed decisions [13]. This study offers the first procedure-specific data on readmissions after VATS wedge resection with a complete ERAS setting. In the present study, the median LOS of one day following ERAS VATS wedge resection was notably shorter compared to the current literature without specific reference to ERAS programs (range from 2 to 4 days) [6, 14, 15], while the 30-day readmission rates following one-day discharge in our cohort (7.8%) were similar to current evidence (7.3%) [6]. Furthermore, the incidence of readmissions after ERAS VATS wedge resection was indeed lower when compared to ERAS VATS lobectomy irrespective of 30- or 90-day follow-up period [9].

In consideration of surgical pathophysiology [1, 16], patients undergoing major surgery, such as pulmonary lobectomy, may exhibit higher levels of preoperative chronic psychosocial stress in comparison to individuals undergoing minor surgery, like pulmonary wedge resection. Intraoperatively, shorter surgical duration for wedge resection may reduce post-anaesthesia adverse events such as nausea and vomiting. Postoperatively, lobectomy is associated with a higher risk of postoperative complications when compared to wedge resection [6, 17]. In addition, duration of chest drainage after VATS wedge resection is shorter as is the incidence of air leak, thereby also giving a lower incidence of pain and pneumonia. Similarly for urinary tract infections, as patients after VATS wedge resection does not have a urinary catheter. These potentially indicate a reduction in readmissions after ERAS VATS wedge resection.

The absence of an increased incidence of readmissions following early discharge is unsurprising [8], as the findings did not identify early discharge as an independent factor for readmission after wedge resection. Recent studies, which have employed large cohorts and directly compared postoperative day 1 discharge, consistently report that the readmission rate did not show any increase [6, 18, 19]. Moreover, there is evidence that implementing ERAS programs for patients who underwent lung resection can reduce LOS, morbidity, and costs but do not impact readmissions [20]. Therefore, greater efforts should be directed toward reducing readmissions by addressing existing risk factors, ultimately facilitating an accelerated patient rehabilitation process following ERAS VATS wedge resection.

As expected, the surgical-related reasons for readmissions after ERAS VATS wedge resection were more frequent during the period from POD 0–30 compared to POD 31–90. A similar tendency was observed in our previous study on ERAS VATS lobectomy [9]. Despite our findings extend to POD 90, pneumothorax and pneumonia were dominant for readmissions after wedge resection, which is consistent with prior studies [21, 22].

The incidence of overall, early and late readmissions due to pneumothorax was relatively low at 2.9%, 2.8% and 0.1%, respectively. Similar outcomes performed in the pneumonia leading to overall (3.3%), early (2.0%) and late readmissions (1.3%). But it is worthy to note that pulmonary complications during the index hospitalization emerged as a predictor for readmissions within the first 90 days after wedge resection. This underscores the importance of vigilant monitoring and follow-up for patients who have experienced pulmonary complications during their initial hospital stay to mitigate the risk of subsequent readmissions. Furthermore, given the potential consequences of postoperative air leak, including increased risks of pneumonia and other complications, as well as patient discomfort due to chest drain placement, it’s essential to optimize current ERAS programs. Strategies, such as selectively omitting chest drains in appropriate patients and considering the use of surgical sealants in high-risk individuals with postoperative air leaks [23–25], could be beneficial. These measures aim to reduce the incidence of postoperative air leak and the need for chest drain placement, ultimately improving patient outcomes and comfort following pulmonary surgery.

Certainly, paying attention to urinary tract infections, pain, and other potential factors related to readmissions is also essential, as indicated by our findings in this study. These variables can impact post-discharge recovery and the likelihood of readmissions. Therefore, they also need thorough consideration and proactive management to enhance patient outcomes.

Significantly, our study has revealed that neurological complications during the index hospitalization can serve as predictors of readmissions. The majority of these neurological complications in our study were attributed to cognitive confusion or delirium. Consequently, it may be beneficial for future perioperative care to refer to current guidelines for postoperative delirium [26]. Additionally, in line with previous studies [14, 21, 27, 28], we found that male patients had a higher risk of readmissions after wedge resection. This information may be applied in preoperative education and individualized therapy plans to better address the specific needs and risks of male patients undergoing this procedure.

The strengths include a consecutive series with complete follow-up due to the national hospital registry system in East Denmark. Every patient in Denmark has a social security number and can be traced accurately in electronic records. Therefore, the validity of the data is very high. However, the retrospective study design is a limitation associated with selection and statistical bias. Secondly, only evaluating surgery-cause readmissions may be an underestimated incidence, however this is the first procedure-specific evidence to report readmissions after ERAS VATS wedge resection, and we wanted to show the specific results directly related to surgery. Certainly, owing wedge resection are always employed for diagnosis and therapy to benign or malignant nodules, future research could include evaluations for completing risks to admission after discharge, thereby improving comprehensive prognosis. Thirdly, we did not exclude patients who died within the first 30 postoperative days (*n* = 2) when computing outcomes for the 31–90-day period. This could potentially introduce statistical bias. Fourthly, the degree of complying with ERAS programs was not evaluated, which may be a confounder for predicting readmissions. A previous systematic review regarding laparoscopic colorectal surgery following ERAS programs indicated that compliance could influence readmission rates [29]. Fifthly, as we have implemented the ERAS programs in our department for over 10 years, we lack comparable data of cases without ERAS programs. However, published data from other institutions [30, 31] suggests that our ERAS program is very effective when compared to standard care.

## Conclusion

Readmissions after pulmonary wedge resection following an effective ERAS VATS setting remain significant, especially due to pneumothorax and pneumonia. Improving current perioperative care may potentially reduce readmissions.
